# Pre-erythrocytic antibody profiles induced by controlled human malaria infections in healthy volunteers under chloroquine prophylaxis

**DOI:** 10.1038/srep03549

**Published:** 2013-12-19

**Authors:** Philip L. Felgner, Meta Roestenberg, Li Liang, Christopher Hung, Aarti Jain, Jozelyn Pablo, Rie Nakajima-Sasaki, Douglas Molina, Karina Teelen, Cornelus C. Hermsen, Robert Sauerwein

**Affiliations:** 1 Department of Medicine, Division of Infectious Diseases, University of California, Irvine, CA 92697, USA; 2 Department of Medical Microbiology, Radboud University Medical Center, Nijmegen, The Netherlands; 3 Antigen Discovery, Inc., Irvine, CA 92618, USA

## Abstract

Complete sterile protection to *Plasmodium falciparum* (*Pf*) infection mediated by pre-erythrocytic immunity can be experimentally induced under chloroquine prophylaxis, through immunization with sporozoites from infected mosquitoes' bites (CPS protocol). To characterize the profile of CPS induced antibody (Ab) responses, we developed a proteome microarray containing 809 *Pf* antigens showing a distinct Ab profile with recognition of antigens expressed in pre-erythrocytic life-cycle stages. In contrast, plasma from naturally exposed semi-immune individuals from Kenya was skewed toward antibody reactivity against asexual blood stage antigens. CPS-immunized and semi-immune individuals generated antibodies against 192 and 202 *Pf* antigens, respectively, but only 60 antigens overlapped between the two groups. Although the number of reactive antigens varied between the CPS-immunized individuals, all volunteers reacted strongly against the pre-erythrocytic antigens circumsporozoite protein (CSP) and liver stage antigen 1 (LSA1). Well classified merozoite and erythrocytic antigens were strongly reactive in semi-immune individuals but lacking in the CPS immunized group. These data show that the antibody profile of CPS-immunized and semi-immune groups have quite distinct profiles reflecting their protective immunity; antibodies from CPS immunized individuals react strongly against pre-erythrocytic while semi-immune individuals mainly react against erythrocytic antigens.

Malaria is one of the most deadly infections in the world with almost 600,000 casualties annually, predominantly in young children and mostly in Africa^1^. The search for a highly protective vaccine has been going on for decades. Although not yet materialized, prospects for a vaccine with partial protection looks promising^1^. Optimism for the conceptual basis of a vaccine comes in part from the knowledge that people living in areas where *Plasmodium falciparum (Pf)* is endemic develop naturally acquired immunity to malaria illness after repeated exposures to the parasite through childhood and adolescence^2^. Antibodies to *Pf* antigens play a critical role in older semi-immune individuals from malaria-endemic regions^3^,^4^,^5^, but identification of antibody specificities has been constrained to relatively few *Pf* proteins made available through traditional cloning methods (<0.5% of the proteome)^6^. Which and how many of the 5,400 possible *Pf* proteins encoded by the *Pf* genome^7^ elicit protective antibodies remains unclear.

To address this important knowledge gap, we constructed a protein microarray using *Pf*-3D7 genomic data which contained 2,320 individual polypeptides representing 1,200 known and hypothetical proteins, or ~23% of the entire *Pf* proteome^8^,^9^. Previously we probed this array with sera collected from 220 Malian children and adults before and after an intense six-months malaria season and showed that a large portion of the *Pf* proteome can be used to probe the complex interface between the parasite and host immune response^2^. Antibody profiles were identified against known and hypothetical proteins associated with naturally acquired malaria immunity. A similar *Pf* protein microarray was used to identify antibody profiles of individuals from Kenya and to profile antibodies from volunteers immunized with irradiated sporozoites and challenged with controlled experimental human malaria infections^10^,^11^.

Recently, we showed that complete protection against an experimental challenge, can be induced in malaria-naïve volunteers using a protocol of ChemoProphylaxis with Sporozoites (CPS)^12^, and that this protection is mediated by a pre-erythrocytic immunity^13^. Exposure to bites from a total of 45 *Pf* -infected mosquitoes induced durable and solid protective immunity against a homologous *Pf*-challenge infection lasting >2 years^14^. In order to better understand the antibody responses induced by this immunization regimen, we developed a new downselected proteome array containing 809 of the reactive antigens recognized in other cohorts^2^,^8^,^10^(Supplementary figure 1). Here we probed this array with plasma specimens from CPS-immunized individuals and compared the reactivities with that of specimens from adult individuals from Kenya with naturally acquired protective immunity.

## Results

**Figure 1:**
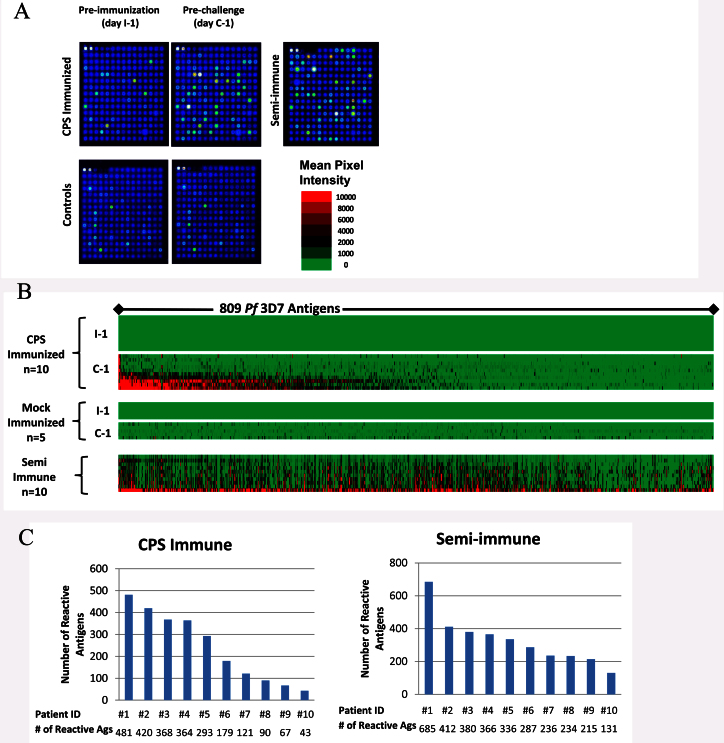
Microarray antibody reactivity. (Panel A). Images of one quadrant (203 of the 809 proteins on the whole array) of arrays probed with plasma specimens taken at different time points. The arrays were probed with Pre-immunization (day I-1) and Pre-challenge (day C-1) specimens from a typical CPS-immunized individual and from one mock-immunized control individual. The semi-immune scanned image represents a specimen from a typical resident of an endemic region of Kenya. The 2 bright spots in the upper left hand corner of each image are IgG positive control standards which react similarly in all specimens. (Panel B). The heatmap depicts the raw reactivity of all individual specimens in this study against all individual antigens on the arrays. The mean pixel intensities for each antigen are recorded in rows and 809 antigens in columns. Red indicates high reactivity, green no reactivity and black intermediate reactivity according to the colorized scale. Since each individual differed in reactivity in the pre-immunization specimen, background preimmune reactivity was subtracted before creating the heatmap. The antigens are sorted from left to right from the most reactive to the least reactive antigen. (Panel C). Numbers of reactive antigens for each of the 10 CPS immunized individuals at one day before challenge (C-1) and 10 semi-immune individuals are shown. Reactive antigens were defined by vsn normalized mean pixel intensity greater than 0.53 after subtraction of the preimmune or USA naive background signals.

Plasma was collected from 10 CPS-immunized and 5 control mock-immunized volunteers bitten by *Pf* un-infected mosquitoes. All CPS-immunized volunteers were fully protected against a subsequent homologous challenge infection while all mock-immunized control subjects developed parasitemia (1). To identify antibody reactive antigens associated with this immunization regimen, plasma specimens taken at pre-immunization (I-1) and pre-challenge (C-1) were probed on the *Pf*- reactive antigen protein microarray. Array images (Figure 1a) showed some background reactivity in pre-immunization plasma (I-1), and a large number of new antibody specificities generated after CPS immunization (C-1). About 24% of the antigens (809) on the array were reactive with plasma from the CPS-immunized group on day C-1 (1 day before challenge). In the mock-immunized control group there was no significant difference in average signal reactivity between days I-1and C-1 (Figure 1a, Supplementary Figure 3). To normalize differences in background reactivity seen among the different immunized individuals, pre-immune background reactivity for each antigen was subtracted from the data in subsequent post-immunization (pre-challenge) time point for each individual (Figure 1b). The breadth of the antibody profile varied among each of the individual subjects, ranging from 481 reactive antigens for the most reactive to 43 for the least reactive (Figure 1c).

To compare Ab specificities between CPS immunized and naturally exposed semi-immune individuals, specimens from 10 adults residing in a hyper endemic region of Kenya were also probed on the same array (Figure 1a&b) showing a different reactivity profile. The breadth of the antibody response among these 10 semi-immune specimens also varied between individuals from 131 to 685 reactive antigens (Figure 1c).

**Figure 2:**
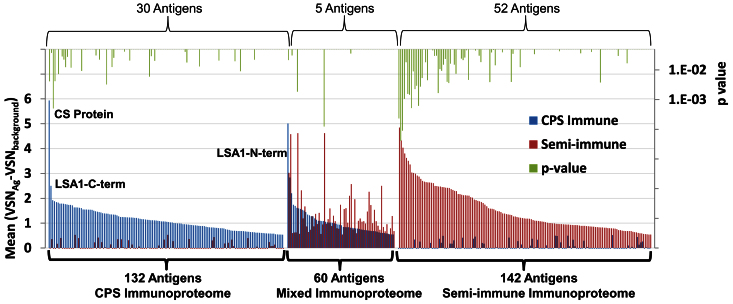
Comparison of the reactive antigen profiles in CPS-immunized and semi-immune individuals. Positive reactive antigens were defined by mean of normalized relative reactivities against the background (vsn normalized pixel intensity with vsn normalized preimmune or USA naive background signals subtracted) greater than 2.0 times the standard deviation of the negative controls, i.e. for this study greater than 0.53. Out of 809 Pf antigens on the array 334 were reactive above the cutoff, 192 in the CPS immunized group and 202 in the semi-immune group. Data show the mean pixel intensities of the reactive antigens from the 10 CPS-immunized individuals at C-1 and from 10 residents of an endemic area in Kenya. 132 antigens reacted only in the CPS immunized group, 142 reacted only in the semi immune group, and 60 antigens were reactive in both immune groups. CSP, LSA1and Rex1 are the most reactive antigens in the indicated groups. Significant Cyber T p values (<0.05) are shown for the comparison CPS immunized vs Semi-immune groups.

To compare antibody reactivity between the 2 groups we calculated the mean signal intensity for each antigen by implementation of variance stabilizing normalization. A quantitative accounting of these differences between CPS immunized and semi-immunes for all of the 334 significantly reactive antigens is shown in Figure 2. A group of 132 antigens only reactive in the CPS immunized group is referred as ‘CPS immunoproteome’. A second group of 142 antigens were only recognized by semi-immune adult individuals from Kenya and is referred as the ‘Semi-immune immunoproteome’. The 60 antigens that were reactive in both CPS- and semi-immune individuals were referred as ‘Mixed immunoproteome’. Reactivity against a total of 87 out of the 334 reactive antigens was significantly different (Cyber T p value < 0.05) between CPS and semi-immunes: 30 in the CPS-, 52 in the semi-immune and 5 in the ‘Mixed immunoproteome’. These results clearly show that the antibody profiles of CPS immunized and semi-immune individuals are remarkably different.

**Figure 3:**
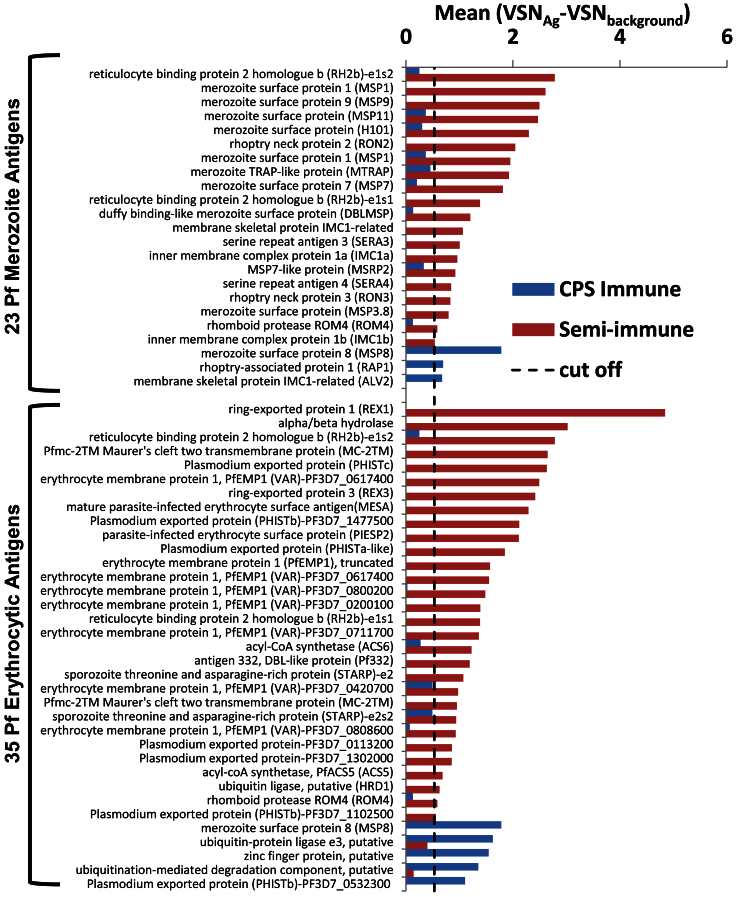
Comparison of the reactive antigen profiles against blood stage antigens in CPS-immunized and semi-immune individuals. To classify the 334 reactive *Pf* antigens in Figure 2, field spaces labeled ‘Gene product’, ‘Gene notes and comments’, and ‘Protein domain names and descriptions’ were searched in plasmoDB; search terms for merozoite proteins were ‘merozoite’ and ‘schizont’, and the terms for the erythrocytic Pf proteins were ‘erythocyte’, ‘ring’, and ‘trophozoite’. This classification identified 23 merozoite antigens and 35 erythrocytic Pf antigens. RH2b-e1s2 (exon 1 segment 2), RH2b-e1s1 (exon 1 segment 1), ROM4, MSP8 were present in both ‘*Pf* Erythrocytic and ‘*Pf* Merozoite categories during the search. Each set of antigens was sorted from top to bottom by mean of normalized relative reactivities against the background in the semi-immune group. Cut off line for reactive antigens is also shown.

Naturally exposed individuals regularly experience high levels of blood stage parasitemia over a period of decades, whereas chloroquine chemoprophylaxis abrogates blood stage parasite proliferation in CPS immunized individuals. So we hypothesized that semi-immune people react mainly to blood stage antigens while CPS immunized individuals will mainly respond to pre-erythrocytic antigens. To categorize the presumed differential reactivity, we identified 2 sets of well-classified *Pf* merozoite and erythrocytic antigens by searching in ‘plasmodb’ (http://plasmodb.org/plasmo/) under ‘gene product’, ‘notes & comments’, ‘protein domain names’ and ‘description’ fields. ‘Merozoite antigens’ had references to merozoite and schizont and 23 of the reactive antigens matched these terms. ‘Erythrocytic antigens’ included proteins with references to ring forms and trophozoites and 35 reactive antigens on the array matched this category. (Figure 3).

Out of 142 ‘Semi-immune immunoproteome’ antigens, 30 are ‘Erythrocytic antigens’ and 20 are ‘Merozoite antigens’ (total 35%). Out of 132 antigens in the ‘CPS immunoproteome’, only 5 could be categorized as ‘Erythrocytic’ and 3 as ‘Merozoite antigens’ (total 6%). Enrichment analysis (Table 1) showed a >6 fold enrichment of ‘Merozoite’ and Erythrocytic antigens” in the ‘Semi-immune immunoproteome’ compared to the ‘CPS immuneproteome’ which was highly significant by the Fisher Exact test. We noticed a significant preponderance of hypothetical proteins (Table 1) (classified as ‘proteins with unknown function’, supplementary Table 1) in the CPS immunized group which is a likely consequence of the paucity of cellular and molecular studies of parasite liver stage in humans. A complete and unambiguous a priori classification of blood stage and pre-erythrocytic antigens is hindered by the lack of proteomic expression data for *Pf* liver stage antigens. Notwithstanding these considerations corroborate the finding that a well-defined set of ‘*Pf*-merozoite’ and ‘*Pf*-erythrocytic’ antigens are well recognized by sera from semi-immune individuals but scarcely seen in the protected CPS immunized individuals.

An alternative to classifying proteins according to life-cycle stage^15^ is offered by mass spectrometry. The classical paper by Florens et al^15^. used multidimensional protein identification (MudPit) mass spectrometry to report evidence of protein expression from merozoite, trophozoite, gametocyte and sporozoite stages of *Pf* 3D7 cultivated *in vitro* in the absence of pre-erythrocytic data. Aligning stage specific MudPit expression analysis with our immunoproteome (Figure 2) did not result in significant enrichment of any parasite stage in either the ‘Semiimmune’ or ‘CPS immuno-proteomes’(Table 1). Furthermore, 30% of all antigens recognized by antibodies in this study were not detected at all in the MudPit study. Clearly, if an immune response is detected against an antigen, it must have been expressed *in vivo*. This discrepancy may be a consequence of differences in the expression profile between parasites propagated in culture vs. *in vivo*, a well-recognized observation for other microorganisms^16^,^17^,^18^. We predicted that CPS immunized individuals would react preferentially to *Pf* orthologs of a set of *P. yoelii* proteins expressed in infected mouse liver cells *in vivo*^19^, but no significant enrichment was observed (Table 1).

**Figure 4:**
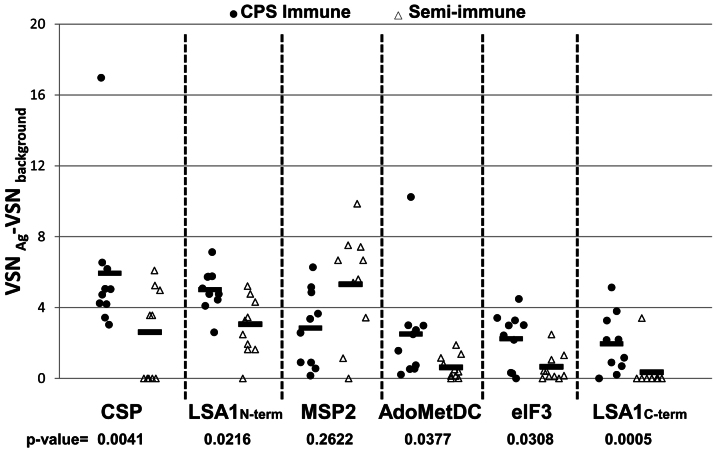
Individual and mean of normalized relative reactivities against the background against the top six reactive antigens in protected CPS-immunized compared to reactivity in semi-immune individuals. CSP, LSA1 N-terminal, MSP2, AdoMetDC, eIF3, LSA1 C-terminal are identified as the 6 most reactive antigens from the CPS immunized group. Next to the individual data of the CPS immunized individuals (circles), the individual normalized relative reactivities against the background of the semi-immune group (triangles) the mean (line) are shown. Five of the six antigens (except MSP2) were significantly more reactive in the CPS immunized group than in the semi-immune group, with p value <0.05.

Figure 4 plots the individual signals from the 6 most reactive antigens from the specimens in the CPS immunized group. The top 6 antigens ranked in order of their mean normalized intensity are CSP, and LSA1 (N-terminal segment), followed by MSP2, AdoMetDC, eIF3 and the C-terminal segment of LSA1. All 6 antigens (or their *P. yoelii* orthologs) have been detected in sporozoites, in mouse hepatocytes from *Plasmodium* infected mice (as *P yoelii* orthologs)^19^ or in human hepatocytes^20^ (Table 2). Five of the 6 antigens showed significantly higher reactivity with plasma from CPS immune compared to semi-immune individuals (p < 0.05) in particular LSA1-N terminal and CSP with reactivity in samples from all CPS volunteers (p value = 0.0216 and 0.004 respectively) (Figure 4). Only MSP2 was not significantly different between these groups. CSP and LSA1 responses in CPS- immunized and primary experimental malaria infections were confirmed by ELISA (Supplementary Figure 4).

## Discussion

This study shows that naturally acquired immunity and CPS-immunity have two distinctly different antibody profiles. CPS immune individuals react to a collection of antigens that are not reactive in semi-immune subjects and vice versa. There is a set of ‘Mixed’ antigens with reactivity that overlaps between the two subject groups. The CPS immunoproteome, lacks reactivity against well characterized *Pf* merozoite and erythrocytic antigens. In contrast, there is a strong blood stage antigen bias in semi-immune individuals.

Some of the protected individuals in the CPS immunized group reacted against hundreds ofPf antigens while others reacted against a few dozen antigens only. However, all of the sera from the protected subjects reacted significantly against only 2 pre-erythrocytic antigens; LSA1N-terminal and CSP. Previously, we found moderate anti-CSP reactivity by ELISA in plasma of this group likely related the use of NANP_6_ repeat protein for coating ELISA plates, rather than the full length CSP which was used here for the microarrays^21^.

The serological profile of Kenyan individuals is comparable to a previous study in Malian children^2^ which showed reactivity against 43/50 of the most reactive antigens reported here. In the Mali study, antibody reactivity against LSA1 N-terminal and full length CSP was greater in asymptomatic than in symptomatic children^2^ suggesting that these two antigens may also be associated with naturally acquired protective immunity. Experimental immunization of human volunteers with radiation attenuated sporozoites delivered by mosquito bites, has also been shown to induce sterile protection against a challenge infection^8^,^9^,^10^,^22^. Antibody profiling with a similar protein microarray, shows a strong association with protection against a panel of 19 antigens including CSP, AMA1 and 16 hypothetical proteins; anti-LSA1 antibodies were only detectable in unprotected volunteers after challenge^10^. The limited LSA1 reactivity compared to our study may be explained by the difference in intra-hepatic sporozoite maturation using the CPS protocol vs radiation attenuated sporozoites. Expression of LSA1 can be visualized *in-vitro* from day 5 post-infection^23^ a few days prior to the appearance of detectable parasites in blood. While complete maturation of liver stages is unabated after CPS-immunization, development of intra-hepatocytic irradiated sporozoites arrests at an early stage before day 5. LSA1 may be an attractive target for induction of protection, although soluble LSA1 immunization failed to induce protection against a human challenge infection^24^.

Understanding the mechanism and specificities of the immune response to the parasite provides guidance to clinical development of malaria vaccines and clinical studies are ongoing to evaluate protective efficacy after immunization with attenuated sporozoites that do not develop asexual parasites. The partial proteome microarray used for this study, which contains 809 proteins, is as we conclude from this study, enriched in blood stage antigens, and liver stage antigens are under-represented. The next generation *Pf* proteome array will contain more than 9,000 features covering all 5,400 encoded proteins and is predicted to uncover more reactive pre-erythrocytic antigens associated with the protective response induced by attenuated sporozoite immunization.

This study shows that antibodies from CPS immunized individuals react strongly against pre-erythrocytic while semi-immune mainly reacts against erythrocytic antigens reflecting their potential as targets for protective immunity.

## Methods

### Specimen collection

Blood samples were obtained from a clinical trial performed at the Radboud University Nijmegen Medical Centre (Nijmegen, Netherlands) from November 2007 to July 2008. The trials were conducted in accordance with Good Clinical Practice and approved by the Central Committee for Research Involving Human Subjects of The Netherlands (ClinicalTrials.gov numbers, NCT00442377 and NCT00757887)^12^. In an outpatient hospital setting of controlled human malaria infections^11^, a total of human 15 volunteers using a standard prophylactic dose of chloroquine, were immunized by exposure to 15 laboratory reared *P. falciparum* or uninfected mosquitoes in 3 subsequent session with monthly intervals (CPS protocol). Plasma samples from each of the 10 CPS immunized and 5 mock-immunized control individuals were taken 1 day before immunization (I-1) and 1 day before challenge (C-1) and stored at −80 C. Individual plasma samples from each individual were analyzed for antibody binding to the *P. falciparum* protein microarray. Kenyan subjects were residents of villages of Museno, Iguhu, Lidambiza, and Shigalagala in western Kenya, high transmission areas for endemic malaria. A serological cross-sectional survey was carried out during the rainy season in June-July 2009. The study which collected 3 ml blood by venipuncture from volunteers, was approved by the Kenya Medical Research Institute Ethical Review Committee [SCC No.1382(N)] and the Institutional Review Board of the University of California, Irvine [IRB 20086148]^25^. Informed consent of all volunteers was obtained before screening.

### Microarray fabrication and probing

A protein microarray of *P. falciparum* reactive antigens (Antigen Discovery Inc., Irvine, CA) displaying sequence-verified polypeptides printed as *in-vitro* transcription translation (IVTT) reactions as described in Davies *et al*^26^. was used. The *P. falciparum* (*Pf*) reactive antigen microarray (Antigen Discovery, Inc., Irvine, CA) contains 809 *Pf* antigens, representing 703 unique *Pf* proteins. The protein targets on this array were down-selected from larger microarray studies^2^,^8^,^10^, based on immunogenicity and antigenicity to humans. Due to gene length, some proteins were printed on the microarray in multiple spots of overlapping polypeptides^8^. The proteins printed on these nitrocellulose coated slides were expressed in cell free in vitro transcription/translation (IVTT) reactions from individual T7 promoter plasmids encoding each protein, and these plasmids encoding each ORF were obtained using a high throughput cloning method previously described^26^,^27^. *Pf* reactive antigen arrays were probed for the N-terminal poly-Histidine tag using a mouse anti-poly-Histidine monoclonal antibody (Sigma, St. Louis, MO) and the C-terminal HA tag using a rat anti-HA High Affinity monoclonal antibody (Roche, Indianapolis, IN). Quality control of array slides revealed over 98% protein expression efficiency of *in vitro* reactions spotted. The primary antibodies were visualized by using the appropriate secondary antibody, Goat Anti-Human IgG, Fcγ fragment specific-Cy5 (JacksonImmuno Labs, West Grove, PA). The arrays were scanned with a GenePix Scanner and quantified using ProscanArray V4 software (PerkinElmer, Waltham, MA). The N-terminal polyHistidine tag was detectable for 778 (96.2%) and the C-terminal tag was detectable for 722 (89.2%) of the *Pf* proteins on the array. Only 9 (1.1%), of the *Pf* proteins on the array were undetectable when probing for either tag (Supplementary Figure 1). The annotated list of antigens on this array is in Supplementary Table 1. Scatter plots in Supplementary Figure 2 show that samples probed on different days and from different batches of chips are highly correlated (R^2^ = 0.79–0.82) indicating that chip printing and probing is reproducible.

For probing, serum samples were diluted 1:200 in Protein Array Blocking buffer (Whatman Inc, Sanford, ME) supplemented with 10% (vol/vol) DH5α *E. coli* lysate (MCLAB, San Francisco, CA) and incubated on arrays over-night at 4°C. Microarray slides were incubated in biotin SP-conjugated affinity-purified goat anti-human IgG Fc-fragment specific secondary antibody (Jackson ImmunoResearch, West Grove, PA) diluted 1:200 in blocking buffer, and detected by incubation with streptavidin-conjugated SureLight P-3 (Columbia Biosciences, Columbia, MD). The slides were washed and air-dried by brief centrifugation. Probed array slides were scanned in a Perkin Elmer ScanArray Express HT at a wavelength of 670 nm, at 95% laser power and 55% PMT. The output grey scale TIFF files generated by the scanner were quantitated using ProScanArray Express software (Perkin Elmer, Waltham, MA) with spot-specific background correction.

### Data analysis

Analysis of the protein microarray data was accomplished following our previously published computational methods^2^,^8^,^10^. Microarray spot intensities were quantified using QuantArray software utilizing automatic local background subtraction for each spot. Each spot on the array is comprised of several hundred pixels, each pixel can have an intensity value from 0 to 65,536. The raw values from array scans were the mean intensities of all the pixels in printed spots after subtracting surrounding background intensities for each protein or negative control. “No DNA” negative controls consist of IVTT reactions without addition of plasmid template. The No DNA spots on each array were averaged and this negative control background value subtracted from every other spot on the array. The variance stabilizing normalization (vsn) method implemented as part of the Bioconductor suite (www.bioconductor.org), performed using the R statistical environment (http://www.r-project.org), was applied to the array intensities. In addition to removing heteroskedacity, this procedure corrects for non-specific noise effects by finding maximum likelihood shifting and scaling parameters for each array such that control probe variance is minimized^28^,^29^. To normalize differences in background reactivity seen among the different immunized individuals, pre-immune background reactivity for each antigen was subtracted from the data in subsequent post-immunization (pre-challenge) time point for each individual. The increase in antibody reactivity occurring after CPS-immunization for each individual is computed by subtracting the background pre-immune reactivity against each antigen from all the post-vaccination time points for the corresponding antigen. Pre-exposure specimens are not available for semi-immune Kenyan individuals who have been exposed to the malaria parasite throughout their lives, so background reactivities from USA naïve individuals were subtracted from reactivities for each semi-immune individuals. Proteins were considered to be seroreactive if mean normalized relative reactivities against the background (vsn normalized pixel intensity with vsn normalized preimmune or USA background subtracted) was greater than 2.0 times the standard deviation of all the negative control “No DNA” spots; so for this study the standard deviation of the negative control spots was 0.265 and the cutoff threshold was 0.53. Differentially reactive proteins between CPS immunized and semi-immune groups were determined using a Bayes regularized t-test adapted from Cyber-T for protein arrays^30^, which has been shown to be more effective than other differential expression techniques, p-value < 0.05 was considered significant. All of the samples in this study were probed at the same time on the same batch of arrays. For ELISA analysis, pre-immune background reactivity for each antigen was subtracted from the data in subsequent time points for each individual. Bayes regularized t-test was calculated and p-value smaller than 0.05 was considered significant (Supplementary Figure 3).

#### P. falciparum gene annotation

Annotation of genes presented in this study follow gene accession numbers published on PlasmoDB (http://plasmodb.org/plasmo/)^31^. ORF ID accession numbers corresponding between PlasmoDB and GenBank databases are presented in the array data file deposited in the GEO Series above.

### ELISA

The ELISA was performed by coating LSA-1 (provided by David Lanar, WRAIR)^23^,^24^ and CSP (from Ashley Birkett, PATH) as previously described^21^. Antigens were coated onto microtiter plates (Thermo Scientific) at 0.03 ug/well for CSP and 0.1 ug/well for LSA-1 in TBS (50 μl/well) overnight at 4°C. The following day the plates were washed four times in Tris Buffered Saline-Tween 20 (T-TBS, USB) and blocked with 300 μl/well casein/TBS blocking buffer (Thermo Scientific) for 1–2 h. Blocking buffer was then decanted and the plates air dried and stored in desiccated foil pouches at 4°C until required for use. For ELISA assay, sera were diluted to 1/200 in blocking buffer supplemented with 10% *E. coli* lysate (Antigen Discovery Inc.) and incubated for 30 min before adding to the plate. Plates were incubated for 1 h with gentle rocking at room temperature (RT), then washed in T-TBS again, and goat anti-human IgG-HRP conjugate (Bethyl) diluted 1/25,000 in blocking buffer was added to the wells and incubated for 1 h at RT. After washing in T-TBS, plates were developed by adding 100 μl/well SureBlue Reserve™ TMB peroxidase substrate (KPL) for 10 min in the dark. Development was stopped by the addition of H_2_SO_4_ and OD read at 450 nm in a Multiskan FC plate reader. The same reactions in blank wells without antigens coated served as negative controls.

## Supplementary Material

Supplementary Information Supplementary Figure 1&2&3&4

Supplementary Information Supplementary table 1

## Figures and Tables

**Table 1:** Comparison of the number of reactive antigens in CPS immunized and semi-immune individuals. The plasmodb fields of Gene Product, Gene Notes and Comments, and Protein Domain Names and Descriptions were searched. The terms used to classify antigens as ‘*Pf* Merozoite antigens’ were merozoites and schizonts, and the terms to classify antigens as ‘*Pf* Erythrocytic antigens’ included references to rings and trophozoites. Mass Spec evidence of expression in different stages was cited from plasmodb.org and published literatures^15^,^19^. The Fisher Exact Test was used to determine significant bias in the reactivities against these antigens when the CPS immunized and semi-immune groups were compared

	Number of Ags* in Group			
	CPS Immune	Semi Immune	Ratio	p value
**Total Ags in Group**	132	142	1.07	--------
**‘Erythrocytic Ags‘(**Figure 3**)**	5	30	6.00	0.00010
**‘Merozoite Ags’ (**Figure 3**)**	3	20	6.67	0.00090
**Proteins with Unknown Function**	57	31	0.54	0.0071
**Other**	67	61	0.91	0.455
**Mass Spec. Evidence^15^**				
**Sporozoite**	56	43	0.77	0.16
**Merozoite**	45	32	0.71	0.12
**Trophozoite**	40	43	1.07	1.00
**Gametocyte**	47	39	0.83	0.32
				
**Mass Spec. Evidence - Py Orthologues^19^**				
**Mouse Liver**	25	26	1.040	1.00
				
Signal P**	28	51	1.82	0.054
**TM**#	40	72	1.8	0.032

*: Ags = Antigens,

**Signal P = Signal peptide,

^#^: TM = transmembrane.

**Table 2:** Annotation of the top 6 reactive *P. falciparum* antigens in CPS immunized individuals

Plasmodb ID	Previous ID	Description	Orthologue in P. yoelii	Infected mouse Liver	human hepatocytes	Mass Spec evidence	Category
***PF3D7_0304600***	***PFC0210c***	***circumsporozoite (CS) protein***	***PY03168***	***Yes***	***----***	***spz, oost***	***Sporozoite***
***PF3D7_1036400***	***PF10_0356 (aa 1-198)***	***liver stage antigen 1 (N-terminal segment)***	***None***	***----***	***----***	***Not Detected***	***Liver***
PF3D7_0206800	PFB0300c	merozoite surface protein 2	None	----	Yes	mrz, rng, szt	Blood, Liver
PF3D7_1033100	PF10_0322	AdoMetDC/ODC	PY04754	Yes	----	spz, tpz	Sporozoite, Blood
PF3D7_1212700	PFL0625c	eIF3 (subunit 10)	PY04546	Yes	----	spz, tpz, gmt, mrz, oost	Sporozoite, Blood
***PF3D7_1036400***	***PF10_0356 (aa 639-1162)***	***liver stage antigen 1 (C-terminal segment)***	***None***	***----***	***----***	***Not Detected***	***Liver***

Spz, sporozoite; oost, oocyst; mrz, merozoite; rng, ring; szt, schizont; tpz, trophozoite; gmt, gametocyte.
