# Supplementary material for: Pre-erythrocytic antibody profiles induced by controlled human malaria infections in healthy volunteers under chloroquine prophylaxis
Source: Sci Rep. 2013 Dec 19;3:3549. doi: 10.1038/srep03549 (PMC4894385; doi:10.1038/srep03549)
Supplement: Supplementary Information — Supplementary Figure 1&2&3&4 [file srep03549-s1.pdf]

Antibody profiles induced by controlled human malaria infections in healthy volunteers under chloroquine prophylaxis

Philip L. Felgner[1], Meta Roestenberg[2], Li Liang[1], Christopher Hung[1], Aarti Jain[1], Jozelyn Pablo[1], Rie Nakajima-Sasaki[1], Douglas Molina[3], Karina Teelen[2], Cornelus C. Hermsen[2], Robert Sauerwein[2*]

[1] Department of Medicine, Division of Infectious Diseases, University of California, Irvine, CA 92697, USA

[2] Department of Medical Microbiology, Radboud University Nijmegen, Medical Centre, The Netherlands

[3]Antigen Discovery, Inc., Irvine, CA 92618, USA

*Correspondence should be addressed to Philip L. Felgner (pfelgner@uci.edu).

Supplementary Figure 1 & 2 & 3 & 4

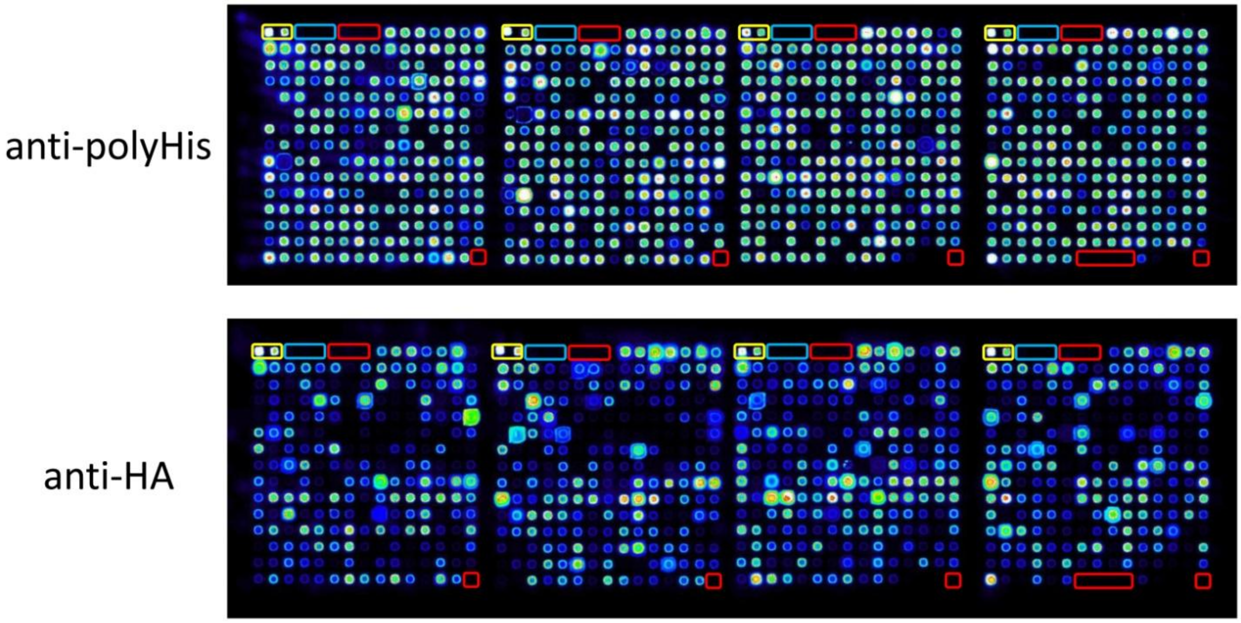

	anti-polyHis	anti-HA	Neither Tag
% Positive	96.2%	89.2%	1.1%

Supplementary Figure 1. Construction of a *Plasmodium falciparum* Protein Microarray. Arrays were printed containing 809 *P. falciparum* proteins, positive and negative control spots. Each array contains positive control spots printed from 2 serial dilutions of human IgG (yellow box), buffer control TTBS (blue box), and "No DNA" negative control spots (red box). The array was probed with anti-His or anti-HA antibody, 96.2% of the protein spots were positive for the His tag, and 89.2% positive for HA tag, contributing to the expression of over 98.9% proteins.

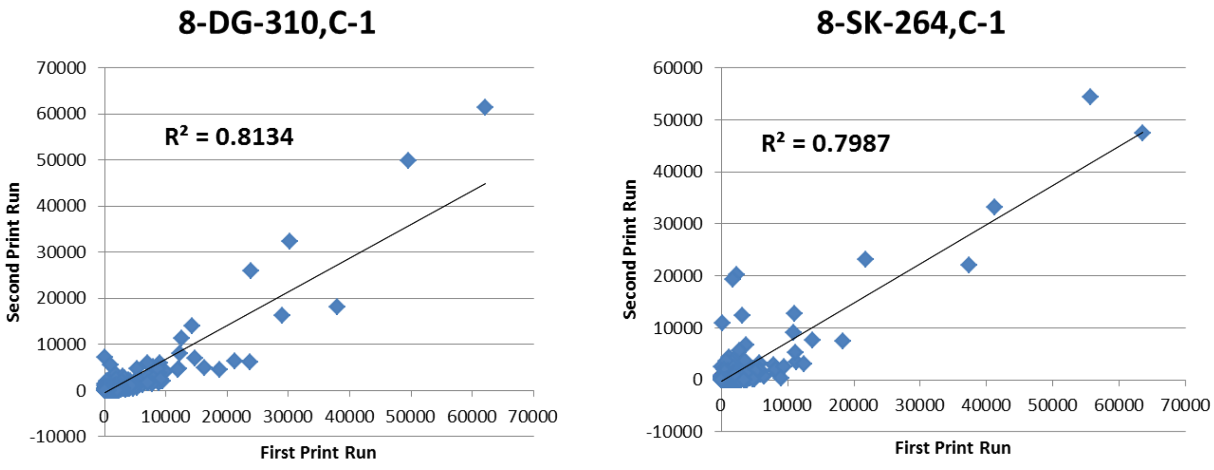

Supplementary Figure 2. *Reproducibility between assays of the same samples done on different days and using different batches of chips*. Two batches of chips were printed several months apart. The results of probing the same samples on the First Batch of chips were compared with results from the Second Batch. These assays were done several months apart. The results between the two assays are highly correlated with R2 values of 0.8.

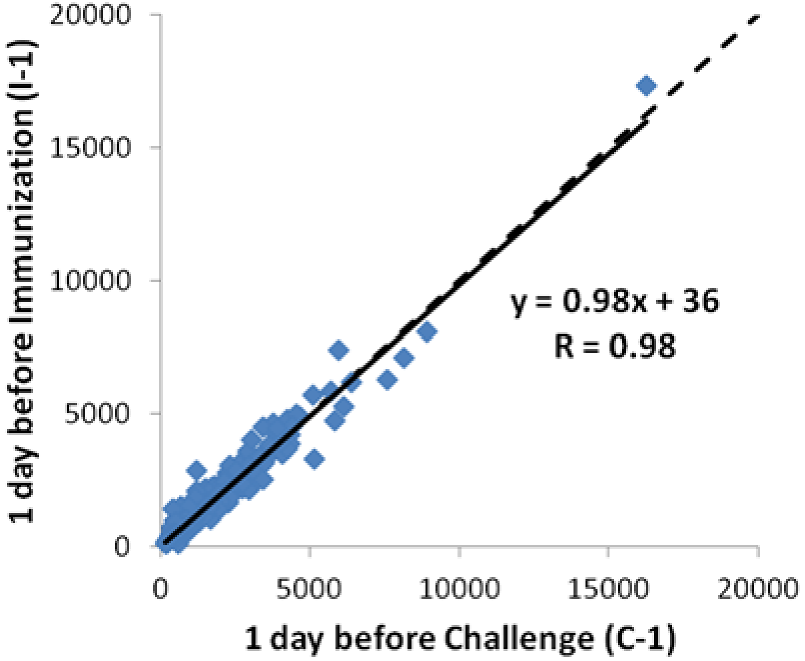

Supplementary Figure 3. *Background reactivity in Mock does not change after exposure to bites from uninfected mosquitos.* The average signal intensities of each antigen were compared at the pre-immune (I-1) and before challenge (C-1) time points in the Mock control group. There was no difference in the background anti-Pf reactivity between these two time points. This indicates that there is no anti-Pf antibody response induced by the bites of uninfected mosquitoes. The highly correlated data also indicates that specimens taken from the same people at different time points and probed on separate arrays are reproducible.

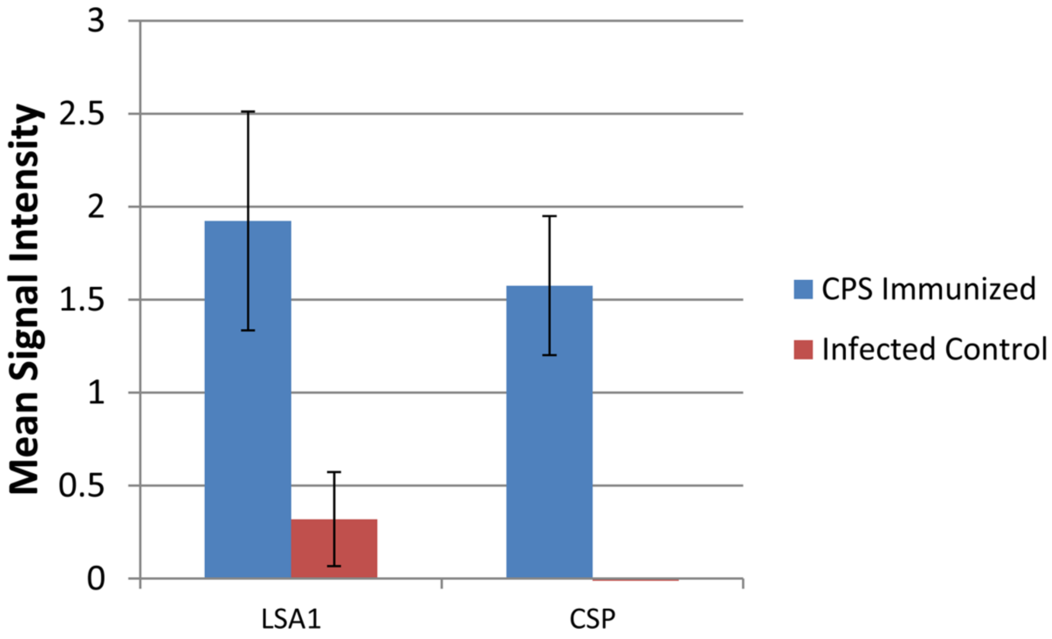

Supplementary Figure 4. Comparing antibody responses associated with CPS Immunization and experimental infection in ELISA. The antibody reactivity against pre-erythrocytic antigens LSA1 and circumsporozoite protein (CSP) are measured in samples collected at one day before immunization (I-1) and one day before challenge (C-1), from both CPS immunized individuals and experimentally infected controls. Pre-immune background reactivity for each antigen was subtracted from the data in C-1 time point for each individual. CS Protein and LSA1 are significantly more reactive in CPS Immunized individuals than in experimentally infected subjects.
